# Scattered Leaves from a Physician’s Diary

**Published:** 1900-07

**Authors:** 


Scattered Leaves from a Physician's Diary. By Albert Abrams, A. M., M. D. (Heidelberg), F. R. M. S., Author of "The Antiseptic Club." St. Louis, Mo.: The Fortnightly Press Co. 1900.

The author of "The Antiseptic Club" has blossomed out as a short story writer in this little work and has given us something of an unusual collection of stories bearing on medical matters and medical men. There is just enough of a scientific flavor about the collection to give them a professional stamp, and as stories, judged from a purely literary viewpoint, they are, in the main, much better than the usual storiette we run across nowadays. Octave Thanet, by all means the most accomplished short story writer of today, has hardly written anything more acceptable than Dr. Abram's "A Mystery of the Latin Quarter," or "The Euthanasia Club."

In these clever stories, Dr. Abrams, with the inborn instinct of the true short story writer, touches all the human emotions from the queerly ludicrous to the tenderly sad, not omitting a flight now and again into the bizarre.

All in all the volume is a highly interesting little collection of stories and is well worth the reading at odd times. N. W. W.

